# Unfinished health systems and policy research agendas for ASEAN Economic Community

**DOI:** 10.1186/1471-2458-14-S1-I5

**Published:** 2014-01-29

**Authors:** Supasit Pannarunothai

**Affiliations:** 1 Faculty of Medicine, Naresuan University, Phitsanulok 65000, Thailand

## 

This Postgraduate Forum was originally a platform for postgraduate students in health systems and policy of Gadjah Mada University, Universiti Kebangsaan Malaysia, United Nations University International Institute for Global Health and Naresuan University to meet annually to exchange their research studies to strengthen their capacities in involving health policies in their countries. In 2013, the forum is taking place at Naresuan University to set the agenda for the 2015 target of economic integration among member countries of the Association of Southeast Asian Nations. The target is imminent, but the groundwork for the health systems and policy research of the AEC is limited. More collaborative works should be mapped to enhance good integration of economic policy with good health impacts especially for the vulnerable groups. More countries are welcome to join the forum for the most attainable health benefits of all members.

